# Draft genome assembly and annotation of the masked birch caterpillar, *Drepana arcuata* (Lepidoptera: Drepanoidea)

**DOI:** 10.1016/j.dib.2020.106531

**Published:** 2020-11-14

**Authors:** Chanchal Yadav, Myron Smith, Dele Ogunremi, Jayne Yack

**Affiliations:** a Department of Biology, Carleton University, Ottawa, Ontario K1S 5B6, Canada; b Canadian Food Inspection Agency, Ottawa Laboratory Fallowfield, Ontario K2J 4S1, Canada

**Keywords:** *Drepana arcuata*, Lepidoptera evolution, Draft genome, Drepanoidea, Social, Larval vibroacoustics, Functional annotation, Gene prediction

## Abstract

The masked birch caterpillar, *Drepana arcuata* Walker (Lepidoptera: Drepanidae), and other Drepanoidea (Lepidoptera) species are excellent organisms for investigating the function and evolution of vibratory communication and sociality in caterpillars. We present a *de novo* assembled draft genome and functional annotation for *D. arcuata*, using a combination of short and long sequencing reads generated by Illumina HiSeq X and Oxford Nanopore Technologies (ONT) MinION sequencing platforms, respectively. A total of 460,694,612 150bp paired-end Illumina and 395,890 ONT raw reads were assembled into 11,493 scaffolds spanning a genome size of 270.5Mb. The resulting *D. arcuata* genome has a GC content of 38.79%, repeat content of 8.26%, is 86.5% complete based on Benchmarking Universal Single-Copy Orthologs (BUSCO) assessment, and comprises 10,398 predicted protein-coding genes. These data represent the first genomic resources for the lepidopteran superfamily Drepanoidea. Although the order Lepidoptera comprises numerous ecologically and economically important species, assembled genomes and annotations are available for < 1% of the total species. These data can be further utilized for research on Lepidoptera genomics as well as on the function and evolution of vibratory communication and sociality in larval insects.

## Specifications Table

**Table:** 

Subject	Insect Science
Specific subject area	Insects, Lepidoptera, Genomics, DNA Sequences
Type of data	Table Figure Raw DNA sequencing reads Draft genome assembly Genome annotation file
How data were acquired	Illumina Hiseq X FLO-MIN106.1 (Oxford Nanopore Technologies)
Data format	Raw – Fastq Analyzed – Fasta, gff
Parameters for data collection	DNA was isolated from an adult male *Drepana arcuata*
Description of data collection	Adult moth was flash frozen in liquid nitrogen prior to DNA extraction; head, abdomen and legs were used for DNA extraction. DNA sequences obtained by Illumina HiSeq X and MinION platforms were assembled using MaSuRCA genome assembler. These steps were done during 2015–2019.
Data source location	Carleton University Ottawa Canada Sample collected at 45.4215 ˚N, 75.6972 ˚W
Data accessibility	Repository name: NCBI SRA, GenBank Data identification number: PRJNA644671 GenBank Accession Number: JACCPG000000000 Direct URL to SRA data: https://www.ncbi.nlm.nih.gov/Traces/study/?acc=PRJNA644671&o=acc_s%3Aa

## Value of the Data

• This article uses both Illumina paired-end and ONT raw reads datasets to construct a draft genome for the masked birch caterpillar, *D. arcuata,* a species used in research on insect sociality and vibratory communication [1]. The study provides a draft genome for a member of the lepidopteran superfamily –Drepanoidea and thus addresses a knowledge gap of genome sequence within the order Lepidoptera [2].
• This dataset will be useful to entomologists interested in genomics, phylogenetics and pest control, and animal behaviourists interested in behavioral genomic studies relating to communication and sociality.
• This draft genome can be used as a reference for future genomics and evolutionary studies of the order Lepidoptera (moths and butterflies). More specifically, these data can be used to test hypotheses on the development, function, and evolution of vibratory communication and sociality in caterpillars and insects.

## 1 Data Description

**Fig. 1:**
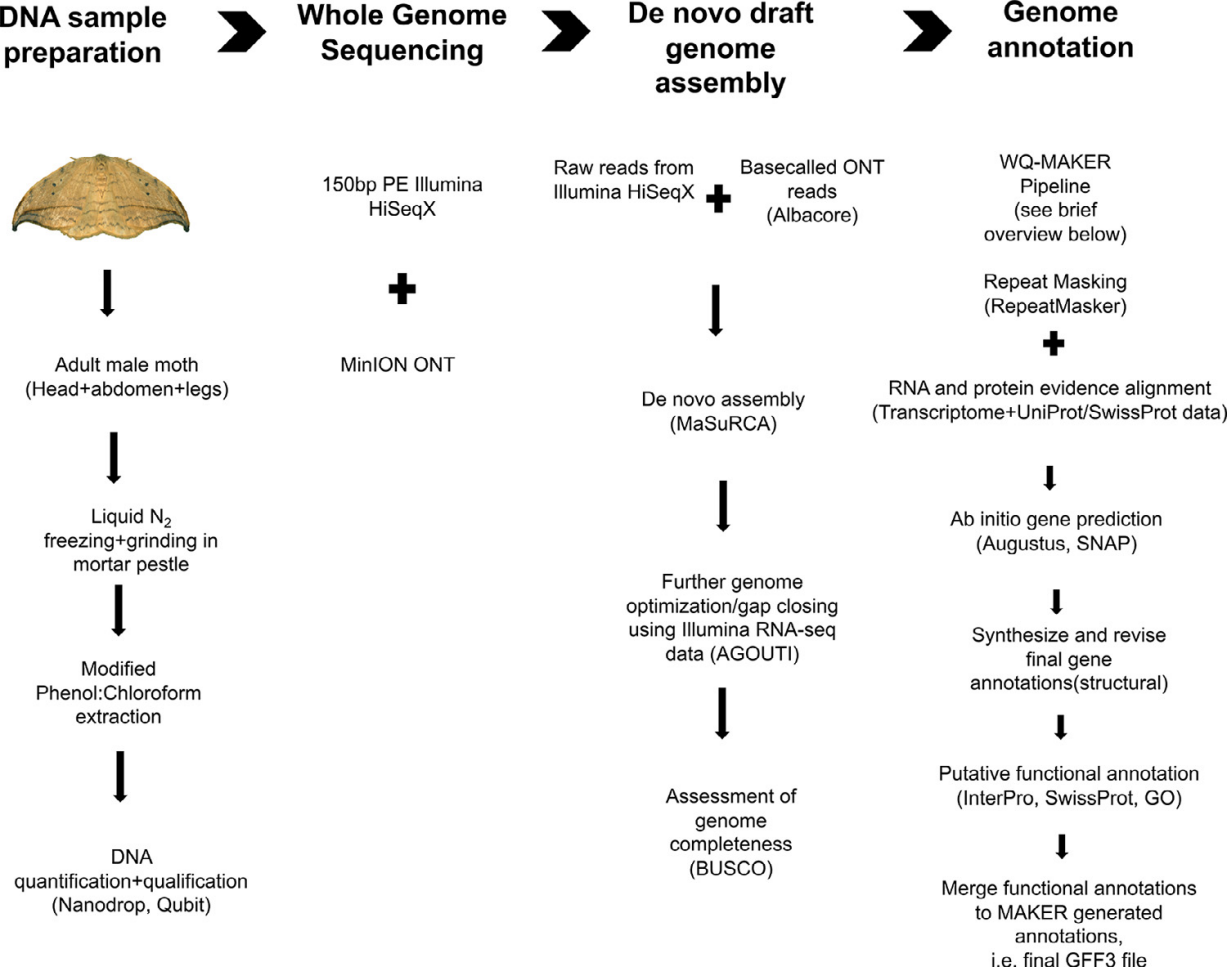
Summary of methods used for draft genome assembly and annotation of *Drepana arcuata.*

This dataset presents the first draft genome assembly with functional annotation for the masked birch caterpillar, *Drepana arcuata* Walker (Lepidoptera: Drepanidae). Raw sequencing data used for genome assembly, and the draft genome can be accessed from NCBI Bioproject PRJNA644671 and supplementary File S1, respectively. Fig. 1 presents an overview of the steps involved in assembling and annotating the draft genome. Taking a hybrid genome approach, both paired-end short reads and long sequencing reads were assembled into 11,493 scaffolds with N50 of 53.8Kb spanning 270.5Mb which represents ∼90% of the estimated genome size [3] (see Supplementary File S1). Supplementary File S1 provides the sequences of scaffolds assembled. A brief summary of statistics on the draft genome and its features are provided in Table 1, and a summary of annotation of genes predicted from the draft assembly is provided in Table 2. Table 3 provides a summary of genome quality assessment performed by BUSCO (Benchmarking Universal Single-Copy Orthologs) [4]. The genome was found to be 86.5% complete based on BUSCO, 10,398 protein coding genes were predicted (Tables 2 and 3) and of these, >84% of the genes were functionally annotated using Blastx 2.6.0+ and InterProScan (Table 2) (also see Supplementary Files S2 and S3). Repeat content was found to be 8.26%, representing the lower extreme of repeat content observed in many other lepidopterans (e.g. 4.7–38%) [5]. Supplementary File S2 provides sequences for putative protein coding genes and File S3 provides annotations done using different databases.

**Table 1:** Characteristics of draft genome assembly and gene predictions for *Drepana arcuata.*

Genome assembly features	
Estimated genome size based on Feulgen image analysis (Mb) [3]	303.18
Assembled genome size (bp)	270,539,787
Number of scaffolds	11,493
Largest scaffold (bp)	503,976
N50 (bp)	53,843
N75 (bp)	26,636
GC (%)	38.79
Heterozygosity (%)	1.05
Number of genes/mRNA	10,398
Number of exons	71,785
Number of introns	60,847
Total genes/mRNA length (bp)	72,086,738
Mean gene/mRNA length (bp)	6590
Mean exon length (bp)	13,856
Mean intron length (bp)	845
Mean CDS length (bp)	1376
% of genome covered by genes	26.6
% of genome covered by CDS	5.6
Mean number of exons per mRNA	7
Repeat content (%)	8.26

**Table 2:** Annotation summary of genes predicted for *Drepana arcuata* draft genome assembly.

Database	Number	Percent (%)
InterPro	8144	78.32
GO	5440	52.31
SwissProt	8774	84.38
Total gene	10,398	–

**Table 3:** Summary of *Drepana arcuata* draft genome quality assessment done using BUSCO v3.0 against Arthropoda orthologs.^a^

Quality assessment (BUSCO)	
Complete	86.5% (single-copy=84.5%; Duplicate=2.0%)
Fragmented	4.2%
Missing	9.3%

a A total of 1066 BUSCO groups were searched.

## 2 Experimental Design, Materials and Methods

### 2.1 Sample collection and sequencing

*Drepana arcuata* eggs were obtained from a wild female caught near Ottawa, ON, Canada, and caterpillars were reared in the laboratory to adult stage. A single male moth was used for DNA extraction and sequencing in order to simplify assembly of a single diploid genome. Wings of the moth were removed and the remaining parts (head, abdomen, legs) were immediately snap-frozen in liquid nitrogen and then ground to a fine powder using a mortar and pestle. DNA extraction was done using a modified Phenol:Chloroform DNA extraction protocol [6]. The extracted DNA was checked for purity and quantity using a Nanodrop 2000 spectrophotometer (Thermofisher Scientific, Waltham, MA, USA) and Qubit 4 fluorometer (Thermofisher Scientific, Waltham, MA, USA), respectively. One µg of total DNA was submitted to Genome Quebec, McGill University, Montreal, QC, Canada, where a 2 × 150 bp shotgun paired-end library was constructed using manufacturer's instructions, followed by paired-end sequencing on an Illumina Hiseq X platform. In addition to paired-end short read sequencing, long read sequencing was performed using MinION sequencing (ONT) at Canadian Food Inspection Agency (CFIA), Ottawa, ON, Canada. Using 2 µg DNA, ONT library preparation was performed using the 1D Ligation Sequencing kit (cat #SQK-LSK108) following manufacturer's instructions. Seventy-five µl of the prepared library was then loaded onto a MinION Flowcell R9.4 (cat # FLO-MIN106.1) according to the manufacturer's instructions and sequences were obtained for 48 h.

### 2.2 Genome assembly and annotation

A total of 460,694,612 raw reads (average quality score, *Q*=36) were obtained from Illumina HiSeqX sequencing, and 395,890 reads (quality score, *Q* ≥ 7) were base-called from ONT (Nanopore sequencing) using Albacore v2.0.2 using default parameters (available at ONT community site, https://community.nanoporetech.com/). Raw reads, without any trimming (as suggested by the assembler), were then used for hybrid genome assembly using MaSuRCA v3.3.1 assembler [7] with the default parameters. *De novo* assemblies generated using MaSuRCA were further optimized for contiguity by using AGOUTI v0.3.3 (Annotated Genome Optimization Using Transcriptome Information) [8] using RNA-sequencing data from NCBI Bioproject PRJNA556910 [9]. The completeness of assembly was evaluated using BUSCO v3.0 (https://busco.ezlab.org) against the Arthropoda database (Arthropoda_Odb9). The draft genome assembly was annotated using WQ-Maker v2.31.9 [10]. In the initial run, RNA-seq transcripts of *D. arcuata* accessed from DDBJ/EMBL/GenBank under accession number GIKL00000000 and protein sequences from UniProt/SwissProt protein database (accessed on May 15, 2020) were used to construct gene models. Repeat masking was also performed during this run with RepeatMasker v4.0.5 using built-in Repbase library [11]. The resulting gene predictions from the initial run were used to train SNAP v2006-07-28 [12] through a second round of WQ-Maker for gene model prediction. Next, Augustus v3.2.2 [13] was trained with BUSCO using the Arthropoda ortholog database and a final round of WQ-Maker was performed with trained SNAP and Augustus for final gene model predictions.

The predicted translated protein sequences were then subjected to functional annotation using Blastp v2.6.0+ against UniProt/SwissProt database (E value cutoff of 10^−6^), and InterProScan v5.26-65.0 for protein domain predictions [14,15]. Detailed information on repeat elements such as DNA transposons, retroelements, and total interspersed repeats was obtained on the final assembly using Repeatmasker v4.0.5 with default parameters and Arthropoda repeat database [16].

## Ethics Statement

Not applicable. No ethics protocols are required for Lepidoptera in Canada.

## Declaration of Competing Interest

The authors declare that they have no known competing financial interests or personal relationships which have, or could be perceived to have, influenced the work reported in this article.

## Appendix Supplementary materials

Image, application 1

Image, application 1

Image, application 1
